# Error in Text

**DOI:** 10.1001/jamahealthforum.2022.5553

**Published:** 2023-02-03

The Special Communication titled, “Graphic Cigarette Warning Labels, the First Amendment, and Public Right to Accurate Public Health Information: Graphic Cigarette Warning Labels Back Under Legal Scrutiny,”^1^ published September 24, 2021, included an error in the text discussion of *Sorrell v IMS Health Inc*. The word “upholding” has been changed to “overruling.” This article has been corrected.
